# ‘It enables the carers to see the person first’: Qualitative evaluation of point‐of‐care digital management system in residential aged care

**DOI:** 10.1111/jocn.16285

**Published:** 2022-03-14

**Authors:** Kasia Bail, Diane Gibson, Alicia Hind, Karen Strickland, Catherine Paterson, Eamon Merrick, Jo Gibson, Maria Kozlovskaia, Amy O’Dea, Bridget Smith, Bernice Redley

**Affiliations:** ^1^ 2234 School of Nursing, Midwifery and Public Health University of Canberra Bruce Australian Capital Territory Australia; ^2^ 2234 University of Canberra Bruce Australian Capital Territory Australia; ^3^ 1410 School of Clinical Sciences Auckland University of Technology Auckland New Zealand; ^4^ Deakin University Burwood Victoria Australia

**Keywords:** dementia, efficiency – organisational, geriatrics, long‐term care, nursing, nursing homes, nursing informatics, patient‐centred care, point‐of‐care systems, quality of health care

## Abstract

**Aims and objectives:**

To evaluate acceptability, efficiency, and quality of a new digital care management system in a residential aged care home (RACH).

**Background:**

Improving care quality and efficiency in RACH, while simultaneously upgrading data management, is a priority for communities and governments.

**Design:**

Participatory action research with mixed methods data collection was employed to evaluate a digital care management system implemented at a 169‐bed RACH. This paper reports qualitative findings of the 2‐year evaluation.

**Methods:**

Qualitative data were collected using focus groups with residents, visitors, nurses, managers, care workers, and consultants; resident/visitor and staff hallway interviews and responses to open‐ended questions in online staff surveys. Data were analysed thematically under the four predetermined study objectives. Reporting adhered to COREQ guidelines.

**Results:**

325 data captures from 88 participants, over seven data sources were coded. Findings indicate that the system was acceptable to both residents and staff due to perceptions of time‐saving and improved quality of care. Increased efficiency was perceived through timeliness as well as reduced time spent retrieving and documenting information. Quality of care was improved through care scheduling individualised to resident needs, with reminders to avoid missed care. Relatives were reassured and activities were scheduled to loved one's preferences. The co‐design implementation process was successful through commitment to quality from leadership teams and prioritising the focus on the holistic needs of the residents.

**Conclusion:**

A strong emphasis on co‐design with care staff in developing and implementing the digital care system contributed to a system that supported nursing and care work, facilitated reporting and documentation, and improved resident care and well‐being including identification of missed care.

**Relevance to Clinical Practice:**

Nurses, carers, administrators, and advocates can support the co‐design creation of information systems that suit the workflow of an organisation and keep the focus on individualised models of care provision.

**Impact Statement**

**What does this paper contribute to the wider global clinical community?**

- Aged care residents have increasingly complex needs, yet, the proportions of nurses onsite have dwindled, and data to inform care quality is underutilised. Mechanisms to improve the efficient use of nursing and care worker time, and maximise care quality, are sought after both for policy and practice.
- This study revealed an improvement in resident‐focussed care, through the development of a co‐designed digital system.
- Enabling nurses to make decisions about infrastructure that supports their work can increase their capacity to meet the individualised needs of residents, while also meeting standardised reporting requirements set by government legislation.

## 1 INTRODUCTION

Increasing delivery of aged care through in‐home care and community support programs means those living in residential aged care homes (RACH) now have higher levels of care needs and more complex care needs than ever before (Gibson, 2020). Along with the growing complexity of health care needs, residents’ care preferences continually evolve over time, underscoring the need for person‐centred care. Care documentation in this setting needs to reflect the holistic care of residents, addressing not only physical care needs but also personal history and psychosocial needs in order to ensure high quality and personalised services (Shiells et al., 2020). Historically, documentation in aged care has failed to meet standardised requirements for personalised interventions (Mariani et al., 2017).

Health information systems that support person‐centred documentation have been slow to develop (Davis et al., 2016; Yu et al., 2020). Digital health information systems offer the opportunity to streamline care documentation and can provide evidence‐informed decision‐making to optimise holistic person‐centred care while contemporaneously capturing data for quality assurance (Stanhope & Matthews, 2019). However, the implementation of health information systems into existing aged care organisations is challenging due to complex governance structures, care processes, and cultural and resource issues (Jiang et al., 2016). To date, there is limited input from aged care residents, care workers, front‐line nurses and members of the multidisciplinary team at the ‘point of care’, and this can hinder usability and acceptability of such systems (Henderson et al., 2016). ‘Point‐of‐care’ systems provide carers with information retrieval and documentation of care at the bedside or other location where care is provided. In this case, where care was convenient to the resident, such as bedrooms and living rooms. Point‐of‐care systems aim to be timely and responsive to the needs of care staff and residents, and this warrants investment and investigation. This paper reports a qualitative evaluation of the acceptability, efficiency, quality, and implementation of a novel point‐of‐care digital health management system implemented as a pilot in a residential aged care service in Australia.

## 2 BACKGROUND

Aged care residents’ preferences for care and support can be highly complex and continually evolve as the person ages. Personalised care plans should be developed in partnership with residents to ensure the residents’ histories are taken into account, and address both physical and psychosocial needs in order to achieve quality care (Shiells et al., 2020). To date, RACH have been unable to provide personalised care plans which impact on the continuity, quality, and safety of care (Mariani et al., 2017). There is evidence that the introduction of health information systems in RACH encouraged residents to share their care preferences, which informed documents such as Advanced Care Directives (Shiells et al., 2020). An Australian study reported that health information systems improved staff perceptions in meeting resident care needs (Zhang et al., 2012). Participants in this study noted benefits due to ease of access to documentation and faster retrieval of resident information which aided a holistic view of the resident. Improved understanding of the resident's wants and needs, particularly for newer staff unfamiliar with the resident, resulted in overall improved care (Zhang et al., 2012).

Nursing RACH documentation provides a record of care information over time (Daskein et al., 2009) and serves as the primary tool for multidisciplinary communication and is therefore the foundation for ongoing resident care (Wang et al., 2011). Documentation provides evidence of the nursing process, and informs quality assurance, legal purposes, research and allocation of resources and funding (Wang et al., 2011). Nursing documentation is also shaped by national accreditation (Australian Commission on Safety and Quality in Health Care, 2017) and registration standards (Nursing and Midwifery Board of Australia, 2016). Ideally, nursing documentation should be aligned with the nursing process of ‘assessment’, ‘planning’, ‘implementation’ and ‘evaluation’ (Cashin et al., 2017; Sanson et al., 2017) to support care delivery. These are important aspects of quality of care.

Frequently, nurses report that documentation is time‐consuming but has a minimal contribution to care delivery, and, when surveyed, nurses state that they prioritise direct care delivery over documentation completion (Ausserhofer et al., 2014). In RACH, the most frequent and time‐consuming tasks are a combination of documentation, medication administration, and verbal communication, which take up more than 70% of nursing time (Qian et al., 2016), with inefficiencies such as duplication of paper into digital systems costing 30 mins per shift in one study (Gaskin et al., 2012). Acceptability of new technology by nurses has mixed responses, often depending on the process of planning, training and support during adoption, in addition to the perceived impact on workflow effectiveness (Ko et al., 2018; Krick et al., 2019).

Globally, the quality and accuracy of nursing documentation is a recognised concern in the provision of residential aged care services (Australian College of Nursing HISOA, Nursing Informatics Australia, 2017, Lee et al., 2020). An evaluation of the content, process, and structure of RACH nursing documentation comparing paper‐based (*n* = 217) vs electronic‐based (*n* = 217) health records found that the electronic records provided a better process and structure for ease of documentation, while the paper‐based records provided more complete and accurate documentation in terms of quantity and quality of information (Akhu‐Zaheya et al., 2018). In another study, both electronic and paper records in Australian RACH were found to be weak in documenting nursing care plans that included measurable resident outcomes (Wang et al., 2015). Given these issues in the literature, further research is needed to evaluate aged care electronic information systems for their efficiency, acceptability and capacity to optimise quality of care.

## 3 METHODS

### 3.1 Design

A 2‐year, three‐stage participatory action research design (Glasson et al., 2006) was used to evaluate the implementation of a novel digital care system at a metropolitan RACH. The setting was a 156‐bed private RACH located in a metropolitan city of Australia. Multiple methods were used to collect qualitative data from residents and staff to evaluate the system and implementation. Qualitative data were collected in August 2020, post‐implementation of the system. This study has been reported according to the Consolidated Criteria for Reporting Qualitative Studies (COREQ) (Tong et al., 2007) and best practices for reporting Participatory Action Research (Smith et al., 2010), (see Supporting Information for completed checklist).

#### 3.1.1 The intervention

The system, ‘ACE (Aged Care Ecosystem)’, focussed on providing point‐of‐care documentation and decision support developed in a co‐design approach with the RACH. ‘ACE’ integrated all communication and information systems in the RACH to minimise the use of paper documentation and streamline operations (work performance and time management). The RACH organisation partnered with Humanetix, aiming to free up staff time to increase direct resident care/contact; improve continuity of care delivery to residents; strengthen compliance with the Department of Health's requirements for documentation in relation to the Aged Care Funding Instrument (Aged Care Funding Instrument (ACFI), 2017); reduce human errors by staff in paper‐based systems and improve the work environment for staff, particularly those from non‐English‐speaking backgrounds. There was no relationship between the research team and the RACH organisation prior to the study commencement.

ACE was adapted from a digital care system designed in the acute hospital setting developed by Humanetix in conjunction with nurses. Trials of the previous system were conducted in Victoria, Queensland, and the Australian Capital Territory (Bail et al., 2020, 2021; Botti, Redley, Considine, et al., 2014; Botti, Redley, Nguyen, et al., 2014; Kent, 2012; Kent et al., 2015). The present study incorporated participatory co‐design methods to progressively adapt and implement the ACE system in the RACH. The project included hardware (swing Personal Computers [PCs] on hallway walls, touchscreen computers at nursing stations, and smartphones), infrastructure (modification of Wi‐Fi, Windows PC settings and memory speed), and future‐focussed enhancements (modifications for easy user administration of passwords and sign in, an active directory for e‐mails, shared device set up and management, business continuity and monitoring, Wi‐Fi and kiosk options) to enable the system to meet the needs of aged care residents and staff.

**FIGURE 1:**
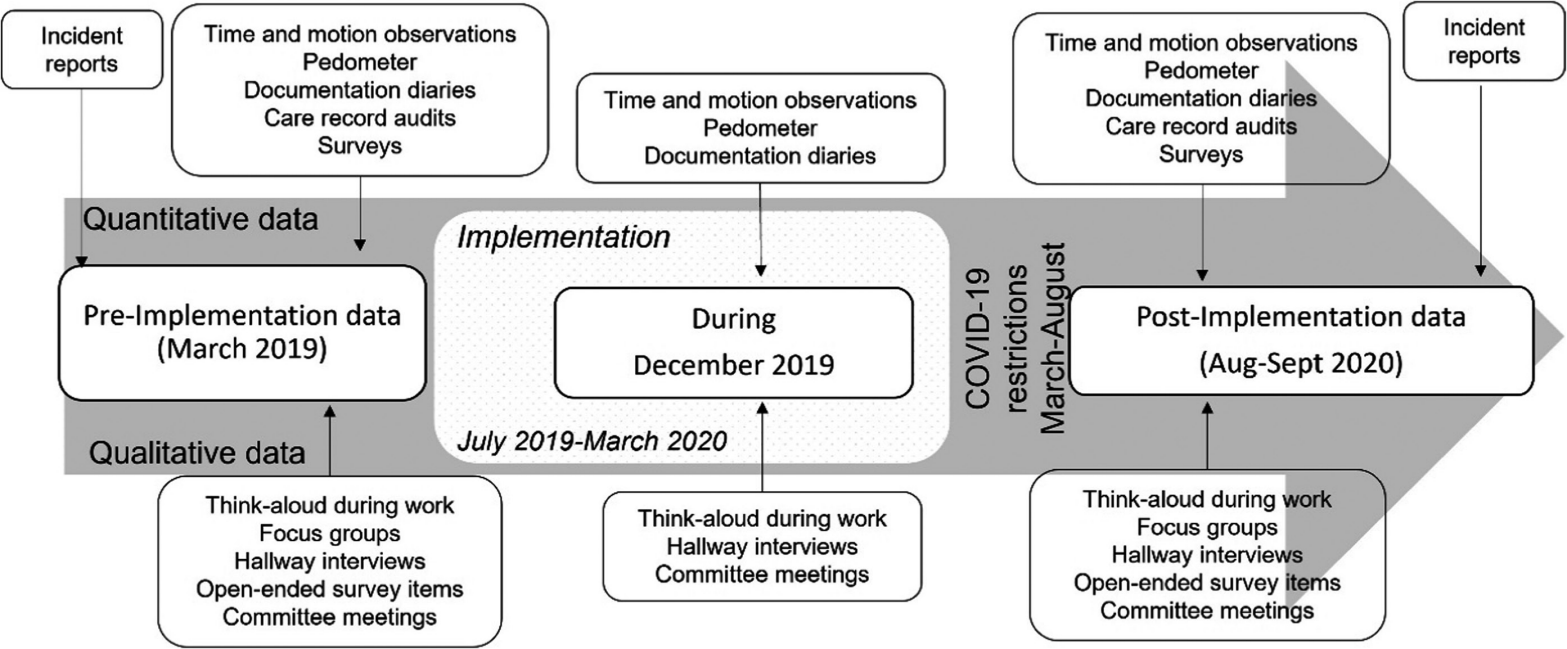
Project timeline and data collection

Co‐design was achieved through cycles of development and implementation of ACE with the RACH over eight stages for a total of 18 months. Action feedback research methods (planned and achieved) for a cycle of feedback included Steering Committee Integration (researcher, participants, product developers); Steering Committee reports, Newsletter updates from Evaluation Committee, Researcher presentations to Jindalee, Member checking of emerging findings. Humanetix responded to feedback about functionality, devices and what was suitable to implement and when. Modules developed within the system included resident assessment and care plans, pre‐admission, and manager reporting. Enhancements included permissions, audit journals, co‐signing tasks, autofill, wing dashboards, nurse reports, and incident management. Figure 1 for the timeline of implementation and data collection, and Table S1 for further details on the Implementation Stages and the ACE product.

### 3.2 Participants

All residents and their visitors were eligible to participate. Residents were excluded if they were unable to communicate verbally or provide informed consent at the time of data collection (e.g. severe hearing and/or cognitive impairment). This decision was guided by facility managers.

All care staff who provided direct resident care, both permanent, casual, and agency were invited via e‐mail and in‐person by researchers to participate in the study. These included nurses (enrolled nurses [EN], endorsed enrolled nurses [EEN], and registered nurses [RN]), assistants in nursing (AINs) (also known as care workers). Other specialty roles invited to participate included nurse educator, clinical nurse consultant (CNC), deputy directors of nursing (DDON), wound nurse, health and leisure staff, and ACFI‐trained care workers. In addition, nurse practitioners, general practitioners, and allied health, such as dietitians, physiotherapists, podiatrists, and speech pathologists involved in care delivery at the RACH were invited.

Information packs that included details about the project and the signed consent processes were distributed to staff members and residents. Study information was also shared via short message service (SMS) (for staff), e‐mail (for residents and relatives), via the RACH newsletter, and posters displayed throughout the RACH. All participants were able to cease their participation at any time. Ethical approval was obtained from the University of Canberra Human Research Ethics Committee (Project #1720).

### 3.3 Data collection

Multiple methods were employed for the qualitative data collection. Using purposive and convenience sampling, 128 participants contributed data to this project. This included 48 residents or their visitors, and 65 staff. People who declined to participate were not asked for their reasons. Of note there was 50% turnover among resident participants (95% due to resident death) over the study period. Data were collected by a multidisciplinary team of 11 health professional researchers (only one was male). All researchers were unfamiliar to residents and staff at project commencement, but their familiarity increased over time as the same staff returned on multiple occasions to collect data. The research team was introduced to staff and residents using participation information materials, newsletters, and in‐person introductions. The diverse research team (registered nurse researchers, professor of sociology, graduates from psychology and dietetics, a postdoctoral sport scientist, and a consumer representative) brought a range of experience and expertise in qualitative and quantitative data collection that provided broad scope for connecting with participants and understanding the varied participant's communication (Smith et al., 2010).

#### 3.3.1 Online staff survey

There were 14 responses to open‐ended questions (i.e. please tell us about your experience with ACE) captured using an online survey hosted on the Qualtrics platform.

#### 3.3.2 Hallway interviews (residents and staff)

Nine members of the research team (KB, BR, DG, MK, CM, AH, BS, BV, and KS) interviewed 45 residents or visitors and 50 staff in locations convenient to the participants. The interviews were frequently in the hallway, or common area, or sometimes at the bedside, and lasted between 5 and 20 min. These were brief conversations with open‐ended questions, for example, ‘tell me what you think about ACE?’, ‘can you provide me with an example of your experience with ACE?’ Responses were captured as field notes by the researcher using an iPad or paper.

#### 3.3.3 Focus Groups (Humanetix, managers, staff, and residents)

**FIGURE 2:**
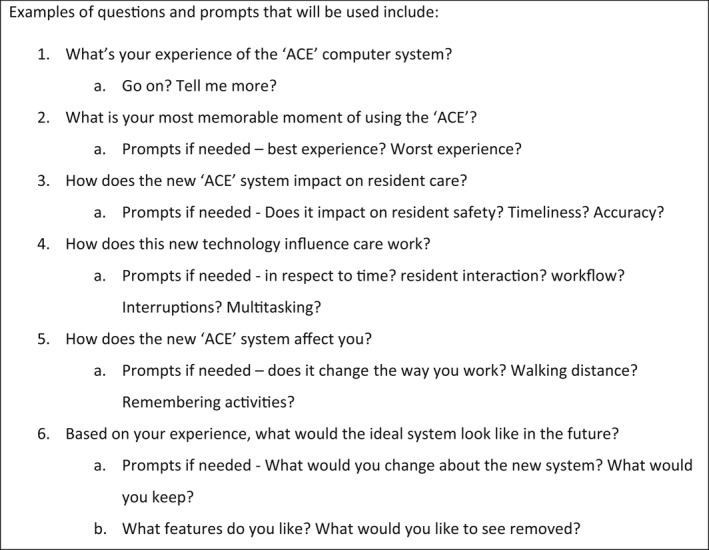
Topic guide

Four focus groups were conducted onsite at the RACH, led by a member of the research team and attended by an observer who collected field notes (KB, KS, BS), audio‐recorded with the permission of all participants, and transcribed for analysis. Focus groups included residents or their relatives (*n* = 4), managers (*n* = 7), staff (*n* = 3), and Humanetix (*n* = 5) and each lasted approximately 1 h. Figure 2 provides an example of a topic guide, which was shared with key RACH staff for feedback prior to implementation.

### 3.4 Data analysis

Data were deductively analysed using the three predetermined study aims of the project Evaluation Framework, namely, A. acceptability, B. efficiency, and C. quality. Interim data analysis following implementation revealed a fourth evaluation area D, the implementation process (Table 1 for framework). Qualitative data from all sources were integrated into Microsoft Excel, and analysed thematically in relation to the framework (Braun & Clarke, 2006; Hackett & Strickland, 2019). At least two members of the research team independently coded all data and interpreted categories. Any inconsistencies and contradictions were resolved through discussion with additional members of the team. Data quality, including any potential impact of researcher bias or assumptions, was enhanced by triangulation using data sources (residents, their visitors, care workers, nurses, managers, Humanetix staff), and different methods of collection and by having multiple analysts examining the data independently (Carter et al., 2014). Analysts included seven researchers (KS, KB, DG, CP, AH, JG, and BS) and one consumer representative (BV). The researchers acknowledged their interest as nurses and health researchers to enable evidence‐focussed aged care, and focussed on achieving broad input from a range of participants to ensure that key concepts were captured, rather than saturation of themes per se (Varpio et al., 2017). Early interpretations of findings were discussed with the Clinical Working Group (which included RACH and Humanetix members), and to a resident morning tea and a staff afternoon tea, as part of the participatory design to enable member checking (Varpio et al., 2017). These events enabled opportunities to ‘pay attention’ to who and how voices and experiences are represented, and to address challenges, pitfalls and limitations as the project progressed (Smith et al., 2010). Data reporting is guided by the COREQ guidelines (Tong et al., 2007).

**TABLE 1:** Implementation Process Framework with qualitative themes and sub‐themes

Aims	Objectives (Themes)	Sub‐Themes
A. Acceptability	1. Reduced time spent retrieving information and documenting care	1.1 Acceptance of ACE system ‐ easy to use 1.2 Contemporaneous documentation at point of care 1.3 Immediate access to information at point of care 1.4 Reduced searching (walking, reading, flicking)
	2. Improved satisfaction of staff and residents with care	2.1 Satisfaction with increased resident safety 2.3 Satisfaction with increased person centredness
B. Efficiency	3. Improved consistency of staff working with management‐approved clinical treatment protocols	3.1 Easy access to most recent specialist review improves staff responsiveness to care changes 3.2 New immediacy of translating care planning update into care interventions for residents 3.3 Easy visibility improves person centred care
	4. Reduced errors by omission and missed documentation	4.1 Improved information capture 4.2 Easy to see information 4.3 Preventing inaccuracies in documentation
	5. Improved management decisions, supported by aggregated data on resident welfare for the allocation of resources	5.1 Flagging enables resource prioritisation 5.2 Operational efficiency potential
C. Quality	6. Improved resident health and quality of life	6.1 Benefits of care scheduling 6.2 Quality of documentation/information supports quality of care
	7. Reduced perceptions of missed care	7.1 Missed or delayed care is now more visible and easier to address 7.2 Missed care is sometimes related to prioritisation 7.3 The prompts reduce cognitive load for nurses/care workers, which reduces missed care
	8. Increased time spent by nurses and care workers with residents	8.1 Time released to spend time with residents 8.2 The system enables more companionable multitasking
D. Implementation Process	9. Implementation Successes	9.1 Co‐design 9.2 Feedback specific to Jindalee
	10. Teething Problems	10.1 Refining the scheduling issues 10.2 Access to on‐the‐job training 10.3 Frustration at learning, slow speed or glitches
	11. Specific Suggestions	11.1 Lack of access by casuals/professionals can increase missed care 11.2 Interconnectivity with other forms of information technology 11.3 Other suggestions

## 4 RESULTS

Following coding and categorisation, 325 quotations were captured. Table 2 shows the distribution of quotations, demonstrating all sections had representative quotes to support the emergent findings, with the most dominant groups being acceptability and quality. Numerical representation of qualitative data is not essential to reporting but can aid interpretation by the reader (Varpio et al., 2017). Key quotations best representing the sub‐themes were used to enable the reader to gain valuable insights for themselves.

**TABLE 2:** Distribution of quotations across themes/aims

Theme	Number of quotations (*n* = 325)	% of quotations
Acceptability	81	25
Efficiency	42	13
Quality	80	25
Teething	33	10
Suggestions	40	12
Implementation	49	15

Note

To make it clear where each quotation comes from, see codes in Table 7.

Each of the three evaluation aims, acceptability, efficiency and quality, are set out in turn below. The fourth section relates to the process of implementation. Each theme is displayed below, with quotation examples supporting the sub‐themes embedded within the evaluation framework (Tables 3, 4, 5, and 6).

**TABLE 3:** A. Acceptability

Theme 1. Reduced time spent retrieving information and documenting care	
SHI 15	It's simple – quick to document and easy to check what others have done. Can read easy, clear notes
MFG 2	So they can just tick it off immediately after doing it
SHI 6	The phone is so handy, we can talk to residents and enter the right information while they talk. Before we had to walk back to the desk and write it then go back to resident. Now we save time, can do it at once, it's so handy
MFG 1	We would normally allocate 20–30 min to do our tick sheets and everything, where now it's 5 min here, it's 5 min there, it's 5 min there
SFG 1	Even a bowel chart, to look at a bowel chart, we would have to – if it was someone on A wing, we'd have to walk all the way up to A wing to just look at the last 7 days of bowels. Whereas it takes us 2 seconds to do it here.
SHI 23	For me gathering information it's amazing. But I'm doing far less steps. Previously, I did 10–15K, now it's 5K. ACE is bad for my arse!! But really good for information
M1	It cut down the time, halved it probably, for when we set up a resident for our ACFI
SFG7	With the coroners, if we've had to supply any paperwork, we didn't have to go to any archived notes and go through it. You can type in the dates of the information they want. It could be a little, it could be a lot, and then just print that instead
RHI 20	They are no longer racing to see residents and racing back to write things down, I’m sure it is much nicer and easier for them
RHI 22	I don't have much to say … but I do notice that they spend an inordinate on the phones, because they have to enter it all
RHI 6	Good ‐ I think it [ACE] has helped with care. (Not much experience with the computer system.) It has made the interactions with the nurses/carers better. If something is wrong with the resident, they have the information at their hands ‐ don't have to search through lots of paperwork. They don't lose any paperwork because it is electronic. This is the ideal system
Theme 2. Improved satisfaction of staff and residents	
SHI 7	Have used other documentation tools and this is very similar. Can't think of anything I don't like about it
SFG 4	All that history of who the person was who, a 90‐year‐old lady [who] once was somebody's little girl, and then she courted this man at a barn dance, the first time she was allowed out … It enables them [the care workers] to see the person [first] and the diagnosis second, to nurse the person, and then the diagnosis and other things we're equipped to manage
SFG 1	Being able to do the care scheduling — so every resident gets a morning visit, morning conversation and there's certain residents who always go to exercises or always go to bingo, so we can schedule in
RFG 4	I get the sense that they have that … because my father changed diet, and I’ve had the sense that the staff know what his diet is, and they will tell him if there's something different on today
RHI 6	I can get out and do walks as that's what I want to do, I’m permitted to do that. I get all the care that is necessary in an efficient and non‐intrusive way
SHI 22	It's dementia nursing, if at first you don't succeed, try, try again! [the resident didn't want to get up/wasn't rousable first try, 10min later was amenable]. Having a shower is a bit of a tumultuous experience for people with dementia. So, they don't really want a shower, you need to persist for a bit but not too much. If they're still resistant go and do something else and come back later

**TABLE 4:** B. Efficiency

Theme 3. Consistency of staff working with management‐approved care plans	
SFG 3	We can easily see when a doctor has last been. So, because we've got the doctor's notes separate, so we can easily see when a doctor has been to see a resident, which is really good
SFG 3	If the dietician comes in today and changes Davina to thickened fluids, she does it on the computer and the strategy goes straight into the care plan, so it's done instantly
SHI 25	When I first started using it, the hair was scheduled at 8am, but how do they know how to do it? How to shower that person? ‘How is Mrs Brown showered – oh look it's just one person, because we've always done it that way. So now we've fixed the instructions, a little tab opens with a summary ‘shower two people, wash hair, get towels ready'. When we were previously still relying on ‘I just know'. If auditors come around and staff are fumbling around trying to find that information, that's not good enough
SHI 26	They like you to do your tasks as you go and that can't happen, you know. And we are regular, we know our residents
SHI 45	Some staff just leave it, a computer won't change that
SHI 30	I think there'd be a moderate group of ‘clicking’ staff, that click no matter what
RFG 4	He would say that maybe 80%, and he's high care, so he's very mentally alert, but he's high care because of his physical needs, and he would say that around 80% [of staff] are very good and then there's about 20% who are a bit impatient or want to do it their way
Theme 4. Errors by omission and missed documentation	
MFG 3	The handwriting is terrible. And I can't talk, my handwriting is also terrible. So being able to read what people have written is wonderful
SS 2	Alerts can be found very easily
SFG 2	Yeah, it's very clear and neat. Everybody will understand the same thing
SS 10	I have been able to filter and look at the clinical notes for residents so that I can catch up on their current health status
SFG 2	Because they are legal documents and they need to be signed
SFG 3	Before we use to write notes in reports and incidents and I honestly feel like I didn't write it correctly, because we come from non‐English backgrounds
Theme 5. Improved management decisions	
SFG 2	I have to follow up on that. So, it just gives you a heads up, which is good
MFG 4	The other thing is we can pick up any issues and deal with them right away, which saves a lot of time. And it means you can go and talk to the nurses about the issue and get it dealt with
SHI 13	But education is needed to flag things, so that they are flagged to me. It's fabulous when I come on in the morning and see wing view with flagged items

**TABLE 5:** C. Quality

Theme 6. Improved resident health and quality of life	
MFG 7	The vision was to have it resident centred, and I really think that that's working
	The staff are able to give the care that the resident actually needs, not what they think they need. So, it's actually telling them, for example, a resident needs to be repositioned, sometimes they will usually do that one, but now it's actually prompting them that, hey, I have to reposition this resident, so this resident has been sitting here for a long time
SHI 40	I think it's very good. It constantly reminds us about things that need to be including toileting. I think it will improve care given to residents
SHI 42	It seems really good, we were having lots of conversation about mum … to make sure all the staff knew the same thing. And they pulled out the [ACE] device and showed me where it all was. It was really reassuring to know that staff knew what things had changed and what mum needed … making sure that she was opening her bowels once a day‐ being aware of. Putting her to bed not too late, she was sleeping in chair and then not being out to bed till 10. Now they do it at 8pm
HFG 2	We're also trying to improve the quality of care and on occasion that means you've got to enforce compliances, and those compliances might be as simple as, and as fundamental as we have a care schedule
RHI 18	The nurses use the system to record everything they do as they do it. Think that the technology has impacted her care for good. I’ve noticed a distinct bettering of the care
RFG 3	That's about the only thing [that's changed with ACE] actually, [they ask more often] “Have you been to the toilet?
RHI 10	I feel things are well organised. One knows what is going to happen
Theme 7. Reduced perceptions of missed care	
SHI 7	ACE is the best tool to remind us to do something, it really helps us not to miss things
SFG 7	I do believe that residents aren't being missed as often, because we've got those prompts that are happening, so, yes, that would equate to an improvement in care
SHI 13	I like it, I can come in and look straight at overdue items ‐ that's the main thing, can see what's done and what's not been done
SHI 33	It's never 0 and it's never 100% that's why it's a 24‐h service. Get everything done to keep people alive and comfortable, but not everything they maybe want. Meds and personal care done but maybe not the washing off the line
SHI 43	Half hour later maybe, but always finalised. You prioritise residents too, some residents come late
SHI 8	We prioritise safety first, paperwork has to be number second. We can always handle it, but we have to change the order of care delivery based on what's happening, which action takes priority. That's why sometimes things are left undone in ACE, not a priority to enter it. Residents change a lot, 1 day fine, next day cranky, changes what we do, and when. But we can always handle it
SHI 1	It's just too busy. We have some aggressive residents so 100% care may not have been given to some residences due to that. Or some sick residents. So not equal care given to each resident
SHI 40	I don't have enough time – that's why care has been missed
SHI 43	You can't be perfect I suppose
SHI 8	It's not perfect, 10 is perfect, not possible
Theme 8. Increased time spent by nurses and care workers with residents	
MFG 4	It gives us more time, if that makes sense, to be with the residents and discuss things with them and talk with them
MFG 1	I have noticed a lot more staff sitting … with residents and actually engaging with them, than I ever used to, doing crosswords and stuff, and I’ve been seeing heaps – even the RNs, when they've got a bit of spare time, they're sitting down with – stuff that I haven't seen for ages… obviously given them a bit more time to be able to spend one‐on‐one with residents, which is good. It's nice to see
SHI 36	Sometimes resident wants to stand up even though they can't, they will fall. That's the main benefit, is the phones, they give me a phone, so now I can sit with the resident and do my notes, and keep them company and supervised and chatting and they are safer than if I was at the nursing desk…. It helps me check things make sure nothing is forgotten. And I can give residents reassurance while I’m documenting
SHI 8	Everyone can see you writing, there's no privacy. If a relative doesn't expect what I’m writing about refusing things, sometimes I wrote about behaviours, to tell RN. Better when can write with more privacy
RHI 20	The interaction is still the same. Technology doesn't impact the care. […] The staff sit in front of me and fill in the information on the phone, I don't mind it, I think it's good
RHI 21	Have seen the nurses use the system in my room at times it is distracting

**TABLE 6:** D. Implementation

Theme 9. Key components of successful implementation	
SFG 2	All the AINs [care workers] have been working so that they do not have to change according to the system. Instead, actually the system is being built to their benefit or how they are or how they work. So it's been built on that, which is really good
SFG 2	I feel really proud that we are participating. Everyone is participating in building the software
SFG 4	I think it's gone beyond what I expected it to. I think it's been really good. I like the way the Humanetix are good to work with, and how they are so understanding and forgiving. So, on week we'll say, oh, can you change that? And the next week we'll say, oh, no, we want it like this. Well, you had it like that last week. But – okay, we'll change it back. (SFG 4)
HFG 2	I actually see that as also a positive because people want improvements in the system and the stakeholder group is just growing now
RHI 2	The staff are good, like part of the family. They know everything about me, what I want. Just got to live day to day, they handle that very well. I think they do very well. My quality of life is better since coming in here. I was going downhill, not leaving the house much. Now I socialise more, better company, my diabetes is better looked after, it's good. They're good to me here
SHI10	They are really good here they try really hard they will take everyone here that others won't take: Severe behaviours, smokers, people with drug and alcohol, weed, homeless. I love it. If I’m dying this is where I want to come. The care is good. They have the longest retaining staff in the whole of Canberra
Theme 10. Teething problems	
SHI 20	It's a new app, in other sites I have used different systems. There are a lot of things we have to do twice. If you are doing personal hygiene, you've got shower, hair, toothbrush, hearing aids. It includes everything, and then you get the same things again another time, everything is again it comes, clean dentures, why I don't understand
SFG 2	We have been through a few hurdles
SHI 28	I use it every day since they started F wing. Formal training hasn't happened, Jane is very busy, I’ve learned from CNC and my colleagues, it's easy, I know most things
SHI 24	[Fiona's] always busy, busy, busy, can't get training. 2 or 3 months
SFI 38	I’m doing notes for g wing [after night duty had ended] because none of the staff were trained, one permanent part time but new, 1 month, and one agency
SFG 1	If I haven't looked at the picture, sometimes I started typing information for one resident into the wrong resident's file, and then realised, oh, I’m in the wrong person. So, I then have to go back
SHI 26	Bugs ‐ clicking activities but they wouldn't show as ‘done'. The stockings were doubled up, now okay, hasn't happened for 2 weeks or so
SHI 44	Only thing that drives me nuts, 1 month ago I wrote lots for the day and it logged me out and I lost everything. I swore a lot
SHI 39	I like it, but it has its issues. We regularly have to refresh as it freezes
Theme 11. Specific suggestions	
SHI 18	Accessing behaviour charts and progress notes helps to know how to avoid getting hit, how to deal with residents. “If he gets aggressive just walk back [out of his room]” isn't enough information. Unfortunately, being agency, you get used to it
SHI 9	Today was the first time he had used ACE, as part of a general round checking on my patients. It was different to other systems I use at other facilities so a bit frustrating to need to learn one more. And remember a new password. Paper is always easier from his perspective because it doesn't rely on finding a nurse. “You can never find a nurse”. With paper folders I can go to the cupboard and find the notes and read what has been happening and then write notes. Now in F wing I can only read my own notes (as the nurses aren't writing in it) so I don't have access to updated information
SHI 57	Most staff haven't got their training and then one staff ends up sitting and doing the ACE while the other ones do the work
SHI 31	The system is very informative for new people ‐ they are less likely to miss everything in there, not like the old folders
SHI 6	I would like a notification of important things, like for those [residents] that walk away get 2 hourly ‘dings’ like a phone [to remind us to check on their location every 2 h]. Another resident has a pressure ulcer, wish we had notifications. BGLs I would like to get an alarm. If they've had high blood pressure, please watch. Absconding is big one. Sometimes we are so engaged in our work!
SHI 6	I would like the phone to interrupt, to notify me, of the really important things like that. Not diet record, not what they had for lunch
RHI 22	Can an individual resident get to see information about themselves? [It would be] good because [then] I can have a good organisation of care

Staff tended to have a mix of reference to Smartcare, Smartward (the name of the acute version of the system) or ACE, and all used them interchangeably in conversation without concern or hesitation. In the quotations, all references to ‘Smartcare’ were changed to ‘ACE’ for ease of reading. Minor editing for clarification and readability is indicated in square brackets []. All names of residents/visitors and staff have been changed to pseudonyms. Sources of quotations are specified, and the key of sources is provided in Table 7.

**TABLE 7:** Source of quotations, with code

	Source of quotation	Code
1	Resident hallway interview	RHI
2	Resident focus group	RFG
3	Staff hallway interview	SHI
4	Staff survey (qualitative component)	SS
5	Staff focus group (mixed: Care Worker, CNC, RN, manager)	SFG
6	Manager focus group	MFG
7	Humanetix focus group	HFG

### 4.1 A. Acceptability

The project's first aim was to evaluate acceptability of the system for residents and staff. There was a high level of acceptability present in the qualitative feedback. The evaluation framework contained two objectives under the aim of acceptability, reducing time spent searching, and improving satisfaction with care.

#### 4.1.1 Theme 1. Reduced time spent retrieving information and documenting care

Point‐of‐care entry is a defining characteristic of the ACE system, and one that was highly valued by staff. Staff's and managers’ comments related to both documentation of care and retrieval of information. For documentation, staff valued the way in which they could document care more contemporaneously than previously as well as the ease of completion (MFG 2; SHI 6; MFG 1). Retrieval of information was supported by the legibility of the documentation (SHI 15), but most particularly the emphasis was on the information being there and accessible when they needed it. Saving time was a recurrent theme (SHI 6; SHI 1; SFG 1) less walking backwards and forwards to document, or to look at records or search through them, and fewer steps. This latter aspect was memorably illustrated by the staff comment that the dramatic reduction in daily steps resulting from ACE ‘is bad for my arse’ (SHI 23).

Another positive aspect related to retrieval of information for external reporting requirements. One manager commented that it had halved the time to complete an ACFI (M1), while another described the ease with which information could be produced for the requirements of the coronial system (SFG 7).

Resident and relative comments reinforced this picture of less racing back and forth by staff (RHI 20). The ready availability of information ‘at their hands’ (RHI 6) was seen to have improved the interactions with nurse and carers, and the electronic system avoided lost paperwork. One resident was less positive, commenting on the ‘inordinate’ amount of time spent entering data on phones (RHI 22), highlighting a potential downside to documentation done via phones and in front of the residents.

#### 4.1.2 Theme 2. Improved satisfaction of staff and residents

In addition to the time saved, there was an accompanying sense from staff of their general satisfaction with the system (SHI 7) and the benefits it brought them in caring for residents. The capacity of the system to provide easily accessible information on individual backgrounds was valued for enabling staff ‘to see the person [first]and the diagnosis second’ (SFG 4). The same aspects were valued by residents and relatives (RFG 4; RHI6). The way in which information can support the delicacies of dignity, where independence is respected, was highlighted by the resident describing a sense of receiving care in ‘an efficient and non‐intrusive way’ (RHI 6). Another benefit of the system was control over scheduling. ACE helped to ensure that residents attended their regular events and received their regular morning visits and conversation (SFG 1) but also enabled flexibility to ‘come back later’ rather than persevering too much at a particular point in time (SHI 22).

### 4.2 B. Efficiency

Within the project's second aim of evaluating efficiency, there were three objectives: consistency of working with protocols (also known as care plans), errors by omission and missed documentation, and improved management decisions. Efficiency was clearly gained through the decrease of walking and searching for documentation, but more systemic effects of management decisions based on improved information oversight were articulated.

#### 4.2.1 Theme 3. Consistency of staff working with management‐approved care plans

The immediacy of access to information translated to more consistent responses in staff to changes in care planning. These care plan changes were at times a general protocol across the facility, but more often in relation to planning for specific resident needs. This could be following review by an external specialist, or a planned assessment by a Jindalee nurse.

Staff highlighted the ease with which they could see ‘*when a doctor has been to see a resident*’ (SFG 3) and also the immediacy of flow through of changes that have been made, as illustrated by the example of the dieticians recommendation for thickened fluids ‘*going straight to the care plan’* (SFG 3). Rather than waiting hours, days, or even reportedly weeks for the changes to catch up with the rhythm of work, the change was much more instantaneous, giving staff ready access to mitigate risks (aspiration pneumonia) and achieve benefits (reducing those risks for the resident).

Another important sub‐theme to emerge related to the *how* of providing care. ACE enabled a positive shift from reliance on ‘*I just know’* to documentation of resident preferences and needs to provide readily accessible answers to ‘*How is Mrs Brown showered’ (SHI 25)*. Some staff, however, felt that the fast task updates interfered with their rhythm of care – ‘*we are regular*, *we know our residents’* (SHI 26). Others commented that a computer system could only go so far (SHI 45) that there would always be a group of staff ‘*that click no matter what’* SHI 30. This view was reinforced by a relative (RFG 4) who described the 80% who are very good and the ‘*20% who are a bit impatient or want to do it their way’*.

#### 4.2.2 Theme 4. Errors by omission and missed documentation

Residents and staff articulated that they perceived decreased errors, and potential for errors, particularly through decreased missed documentation and improved information capture.

The ease of seeing information was an important component, whether because of legibility (‘*being able to read what people have written is wonderful’* (MFG 3) or the clear formatting of alerts and structure that made information more logically findable (SS 2, SFG 2).

The capacity to tailor information to specific needs, such as filtering to look at clinical notes (SS10) was appreciated, as was the automatic inclusion of names and roles that is part of the system, readily meeting legal requirements (SFG 2). Some staff highlighted that they valued the computer autocorrection for spelling and grammar ‘*because we come from non‐English backgrounds’ (SFG 3)*.

#### 4.2.3 Theme 5. Improved management decisions

Part of Efficiency is the use of system‐level decision‐making based on new access to data about residents in order for staff and managers to better plan their resourcing and prioritisation of care.

The system supported picking up on issues and dealing with them right away (SFG 2, MFG 4). The managers also commented on the cultural shift that was occurring, including education for staff on how to flag things to alert the managers or CNCs. One manager described as ‘*fabulous’* the mornings when she would come in to ‘*see wing view with flagged items’* (SHI 13).

### 4.3 C. Quality

Quality of care was an important aim for staff, residents, relatives, and Humanetix (the product developers). Throughout the quotations, the focus on achieving quality care, and the satisfaction it provides when it is delivered, were clear — in the words of one manager ‘*the vision to have it resident centred’* (MFG7). This section articulates key benefits that staff and residents described related to the three objectives or improved resident health and well‐being, reduced perceptions of missed care and increased time spent by staff with residents.

#### 4.3.1 Theme 6. Improved resident health and quality of life

A range of sub‐themes were clear within the broad rubric of improved resident health and quality of life, and these related to the benefits of care scheduling; the quality of documentation/information supports quality of care; the improved sense of safety for residents; and the improved capacity for managing the delicacies of resident dignity.

Managers felt that care scheduling was important to improving quality of care by aligning care more to the actual needs of the resident and providing prompts in relation to straightforward tasks such as in the example of repositioning a resident. The manager comments that staff ‘*will usually do that one*, *but now it's actually prompting them that*, *hey*, *I have to reposition this resident*, *so this resident has been sitting here for a long time’* (MFG 6). Staff comments supported this view around scheduling, referencing an example around prompts for toileting and the potential to improve care (SHI 40).

A relative described the value of care scheduling in relation to her mother getting to bed in a timely way, and also the reassurance provided by ACE ‘*that staff knew what things had changed and what mum needed’* SHI 42. Another relative made a direct connection between the better documentation, and the role of technology in producing ‘*a distinct bettering of care’* (RHI 18). Other residents and relatives may not have noticed specific changes, but were reassured by a sense that ‘*things are well organised’ RHI 10)*. However, not all responses were positive, with one resident commenting that the only change was being asked more often *‘Have you been to the toilet’* (RFG 3).

The Humanetix product team members were very aware of the role of their technology in providing a care schedule that was part of their key goal to improve quality of life and quality of care for residents. They were ‘*trying to improve the quality of care’* and that might be ‘*as fundamental as we have a care schedule’* (HFG 2).

#### 4.3.2 Theme 7. Reduced perceptions of missed care

Missed care was perceived to have reduced, because (1) tasks that were needed were flagged and ‘flashing in their face’; (2) it was more easily seen when tasks were delayed or missed, and because there was a mechanism to follow up those tasks.

Staff described the scheduling as key to making care happen as planned and making it easier to know what to *do* and not miss items of care (SHI 7, SFG 7). When care items were overdue, they showed in orange and were raised in the hierarchy or visibility in ACE, and this was valued by staff because they could ‘*come in and look straight at overdue items’*, seeing what has been done and what has not been done (SHI 13).

Despite the value attached to scheduling, staff were also pragmatic about the practicalities of prioritisation of care. Part of the hallway interviews was to ask the staff to rate the proportion of missed care. Staff would justify why they had indicated 5%–10% of care was missed, explaining that they had to re‐prioritise based on other needs of other residents. This was summed up by various staff members as relating to the nature of the care task *‘meds and personal care done but maybe not the washing off the line’* (SHI 33), relating to the needs of the specific resident (SHI 43) and also to the principle of care ‘*safety first*, *paperwork has to be … second’* (SHI 8). There was also missed care comments related to sheer availability of time (SHI 40).

These comments highlighted that documentation, including ACE entry, may be appropriately deprioritised based on resident needs, or that some resident needs had to be prioritised over another, based on characteristics such as aggression and level of sickness on a given day (SHI 1).

Staff responses on missed care also raised the issue that just because all tasks were completed did not mean that a resident's needs were all met. This shows an insight into staff perceptions, where they aim for high‐quality standards but do not expect to be ‘*perfect’* (SHI 43, SHI 8).

#### 4.3.3 Theme 8. Increased time spent by nurses and care workers with residents

Multiple staff and residents commented on increased time spent in one‐on‐one time with residents. Managers similarly felt that the new system was giving staff more time to spend with residents (MFG 4, MFG 1). Intriguingly, one manager describes seeing RNs sitting down with residents as ‘*stuff that I haven't seen for ages’ (MFG 1)*, in the broader context of more staff sitting *‘with residents and actually engaging with them’*.

Staff also noted that they could spend more time with residents while multitasking. This was perceived by the staff as an appropriate kind of multitasking, where they were documenting into ACE on their phone, while sitting with residents in a companionable manner, ‘*keep[ing] them company and supervised and chatting and they are safer than if I was at the nursing desk’* (SHI 36). This was seen as reassuring for residents, as well as an increased level of comfort for staff.

One staff member did express concern about writing some content in open spaces, but also recognised the flexibility to choose where they documented with the ACE handheld devices (SHI 8). Staff also indicated that having multiple hardware options (i.e. smartphones, tablets, desk, and wall PCs) meant that they could tailor the hardware and the location to suit the content they needed to document.

Most residents did not find the multitasking problematic once they understood what was occurring on the ACE smartphones (RHI 20). One resident did comment that seeing the nurses use the system in the resident's room was ‘distracting’ (RHI 21).

### 4.4 D. Implementation Process Framework

This was a study of the effects of ACE software on acceptability, efficiency, and quality. It was also a study of the process of implementation. Three main themes emerged from the qualitative data concerning the implementation process: teething problems experienced during implementation but which appeared to be resolved, specific suggestions for future aspects of roll out (which may already have been addressed by the time this report is finalised); and successful aspects of implementation. These were particularly in relation to the satisfaction with the co‐design process between Jindalee and Humanetix; and the satisfaction with the quality of care at the home itself. These warrant attention as they are clearly considered important aspects of successful care by staff and residents.

#### 4.4.1 Theme 9. Key components of successful implementation

Two major sub‐themes emerged in relation to the success of the implementation. The first of these was the co‐design process that was integral to the roll out and development of the ACE system at Jindalee, and the second was the sense among staff, residents, and relatives that Jindalee was a high‐quality service.

The co‐design was seen as key to the success of the implementation, in that it was a shared project with shared goals, focussed on staff workflow as well as resident needs. This created a feeling of ownership and pride among staff at all levels (including care worker as well as nurses) that this was a system that ‘*is being built to their benefit or how they are or how they work’* (SFG 20). The sense that the system was developed for and with the staff rather than on them was clearly core to implementation success for Jindalee staff and Humanetix alike (SFG2, HFG2), as was the way in which Humanetix were so ‘*understanding and forgiving’* in relation to staff feedback (SFG 4).

The quality of care was a recurrent theme among staff, residents, and relatives. Aspects such as the knowledge of individual resident needs and the support for improved quality of life (‘*My quality of life is better since coming here*. *I was going downhill’* RHI 20) and staff perceptions that ‘i*f I’m dying this is where I want to come’* (SHI 10) contribute to an environment conducive to successful implementation.

#### 4.4.2 Theme 10. Teething problems

A range of comments were related to issues that arose as part of step‐by‐step implementation. Staff had different experiences through different stages of the project, and their comments reflect different stages in the process. Three sub‐themes here related to scheduling issues intrinsic to the roll out, access to on‐the‐job‐training and learning the new system, as well as system glitches.

Not all comments were unequivocally positive, and perhaps the most common niggle related to repetition in the system which was occurring at one stage— ‘*and then you get the same things again another time*, *everything is again it comes*, *clean dentures*, *why I don't understand’ (SHI 20)*. Staff did recognise that these ‘*hurdles*’ were part of the process of co‐design (SFG 2), and as noted under Theme 9 above the value of co‐design to staff was widely recognised.

While Humanetix provided a full year of training to all staff, subsequent training was undertaken ‘on‐the‐job’. Staff did report issues related to on‐the‐job training, and while some issues resolved over time and completion of roll out, there were pressure points that emerged particularly in relation to staff turnover (SHI 38). Having one staff member manage the training has the advantage of consistency but increases pressure on key personnel whilst also creating a bottleneck for staff accessing training and log ins (SHI 24). The time pressure on training led to frustrations, but also demonstrated that it was relatively easy to learn (SHI 28). There were, of course, frustrations for some in learning the software (SFG 1).

Some experiences relating to glitches and system slowness were either ‘one‐offs’ (SHI 44) or temporary at certain stages of system development (SHI 39). There were some comments about speed of the system (SHI 26), which were resolved, but highlighted that there could be a concern in future implementations for older buildings with poor internet connectivity. Staff highly valued speed for effective working.

#### 4.4.3 Theme 11. Specific suggestions

Staff and some residents made specific suggestions in relation to the program that warrant consideration. It should be recognised that many suggestions were picked up and considered in the implementation process by the Clinical Working Group and by Jindalee management. Some of the suggestions may have been decided against, or have specific strategies in place, but a mix are provided here ensuring the research provides a voice for all participants.

The most common suggestion for the future is related to access by agency and external staff. Some comments indicated missed care related to agency staff, others to the issues of not being able to access the information when needed (SHI 9). From the agency care worker perspective, access to behaviour charts ‘*helps to know how to avoid getting hit*’ (SHI 18).

Staff raised concerns about the integrity of data entry when the work arounds for agency staff not having access were used. Commonly, this meant writing on scraps of paper, or using someone else's log in, or one person doing documentation while the other person did the work. Staff outlined the risks associated with each of those workarounds (SHI 57). However, where access was available, staff commented on its value in being ‘very informative for new people’ and reducing the likelihood of missing key information (SHI 31).

Another set of suggestions related to the use of notifications and flagging. Different styles of notifications were suggested, often in regard to things that were resident specific, such as reminders to do a 2 hourly location check on residents that walk away, or to prioritise checks on a resident with a pressure ulcer (SHI 6). One resident asked about access to see their own information regarding this as a way of monitoring their own quality of care (RHI 22).

Overall, the qualitative data highlights the experiences of the Jindalee care staff team, the residents, family members, and the Humanetix team. The data have captured the essence of the experiences of care management and delivery using the ACE system, being cared for and the process of developing and implementing the system. The quotations from the participants provide authenticity to the study evaluation by giving voice to those using the system as well as to the residents and their visitors.

## 5 DISCUSSION

The qualitative findings identified that the digital care system in RACH was acceptable, efficient, and enabled quality care with high levels of satisfaction among key stakeholders. In relation to the aims of this study, specifically the system was: A. acceptable, particularly in relation to the time‐saving nature of the ACE system; B. efficient, with reduced time spent retrieving information and documenting care, as well as the timeliness of documentation, and C. enabling of quality care, with ACE facilitating improved contemporaneous and detailed documentation that improved satisfaction of staff with care delivery. Importantly, staff found it easy to plan care and identify any missed care among residents. Of note, was the prioritisation of care quality: staff expressed that the system could not be efficient or acceptable unless it was improving quality first and foremost.

Other research has found mixed results regarding the effect of health information system implementation on nurses’ documentation. Some studies found that documentation time decreased whereas others found the opposite (Ko et al., 2018). The evidence from this study indicates time spent documenting has decreased whilst the overall quality and contemporaneous nature of the documentation has improved. As there is a global concern surrounding the quality and accuracy of nursing documentation within RACH, this improvement in documentation quality is noteworthy (Australian College of Nursing HISOA, Nursing Informatics Australia, 2017, Lee et al., 2020).

Improvement in the quality of nursing documentation within RACH is a significant finding because documentation informs numerous important functions, such as, providing quality assurance, informing legal requirements for care, research, and appropriate allocation of resources (Wang et al., 2011). Of clinical significance, the improvement of nursing documentation has the potential to positively influence aged care as a whole. The effective utilisation of the ACE system provides the opportunity to successfully evaluate nursing care and resident outcomes.

The benefits of having electronic prompts for documentation requirements as well as assistance with spelling and grammar may increase staff members’ confidence with their documentation, and potentially impact staff work satisfaction. This study found that implementation of ACE increased the legibility of documentation as well as the ease of access to information. This finding is supported by other research which found that increased documentation legibility and accessibility associated with electronic documentation led to the delivery of better quality care for nursing home residents (Ko et al., 2018).

Quality of care was an important aim for staff, residents, and Humanetix, with the data supporting that the ACE system allows for care scheduling that was individualised to the residents’ needs, with reminders ensuring care is not missed. Data from the relatives also supported that the quality of care seemed to improve, perhaps by the reassurance of activities being scheduled to their loved one's preferences. Also highlighted was the clear commitment to quality from the Jindalee team and the Humanetix team who were focussed on ensuring that the ACE system supported the care needs of the residents, with improvements to the system negotiated and implemented.

These findings are consistent with previous research which found that when there was an improvement in the understanding of a resident's personalised needs and wants that the care the resident received was also improved (Zhang et al., 2012). The use of the ACE system supported the timely development of personalised care plans for residents which met their holistic care needs by including their unique physical and psychosocial needs, as well as their history and personal preferences for care (Shiells et al., 2020). This leads to the possibility of advancement in quality, safety, and continuity of care, which historically has seen gaps in aged care homes due to the lack of personalised care plans (Mariani et al., 2017). A systematic review highlighted that the ease of data entry and the improvement in quality of documentation detail is key for improving time management with new information technology, but that if the quality of the detail is poor, then the all‐important care improvements do not occur for residents (Meißner & Schnepp, 2014).

The importance of the implementation process was a clear theme that was not an original component of the Evaluation Framework. The data were rich in discussions from both Jindalee staff and the Humanetix team with the co‐design process between Jindalee and Humanetix and the collegial relationships particularly appreciated. Issues that arose for the users of the system appeared to be resolved in a timely way with specific suggestions for future aspects of implementation being taken on board. This positive working relationship between the developers and users significantly contributed to the overall achievement of the three overarching project aims. The co‐design was likely to be the key contributor to avoiding the common unintended adverse consequences noted in other studies (Yu et al., 2013). Research has shown that failure to include stakeholders in the planning stages and providing inadequate training for the implementation of health information technology are major barriers to successful implementation and improved quality of care (Ko et al., 2018). These major barriers were avoided in the ACE implementation through the use of a robust co‐design process supported by the RACH, Humanetix and the evaluation team.

The co‐design process is recognised as providing benefits for design projects themselves, the stakeholders and the participating organisations (Iedema et al., 2010). Co‐design enables an enhanced product to be developed as there is an understanding of what the end‐user needs which is automatically addressed in the design phase rather than having to retrofit feedback later on during the testing and implementation phases. The 2‐year process with regular Clinical Working Group meetings and a clear pathway and process of testing and feedback enabled shared understanding through direct and indirect support, and simultaneously enablement of iterative change in the complex system (Papoutsi et al., 2020). Importantly, this enabled staff, particularly the Nurse Educator, to use training in the tool to modify culture and entrenched practices to improve the quality of care in line with service requirements.

Improved data collection by care staff will provide future opportunities for research to examine key workflow improvements, such as how ‘flagging’ (where the AINs, ENs and RNs can highlight a change in resident status for review and escalation) can be best used to manage resources and staff attention and what information is needed at the bedside to support decision‐making in line with current evidence.

This study was limited to a single site, was quasi‐experimental with a focus on the co‐design of the intervention and implementation of the product, as well as on the intervention outcomes. This paper focuses on the qualitative findings. Further studies which can provide control or cohort groups may elicit further comparative data for improved level of evidence for reliability, and larger participant numbers with multiple sites would enable greater examination, particularly in regard to quantification of efficiency which is needed in this field (Krick et al., 2019). Qualitative research limits generalisability; however, it is notable that the perception of residents and staff on the impact of the technology implementation upon quality of care can only be elucidated qualitatively. In particular, participants in this study highlighted that interpreting efficiency and effectiveness are dependent on improving the quality and access to information about resident needs. Therefore, we recommend that any further research examining efficiency and effectiveness of digital systems in aged care focus simultaneously on examining quality of care, which is not always considered in evaluations (Tubaishat, 2018).

Future implementations and research into technology in aged care can benefit from recommendations arising from this research. Given some concern and confusion by residents and relatives about staff being ‘on their phones’, participants recommended that phone cases identifying the workplace be utilised to aid enculturation of the new method of documentation to be more recognisable as a ‘work’ rather than personal device. Future research can focus on utilising data generated by these kinds of care technology that is a by‐product of care delivery to explore clinical decision‐making, streamlining nursing and care workflow, and examine decision aids to support nurses and carers in providing individualised care with residents and their loved ones.

## 6 CONCLUSION

The improvement in care quality seen in this study was the highest priority for residents and staff, and any satisfaction with the acceptability and efficiency of the system was based on perceived improvement in quality first and foremost. The increased detail of, and access to, resident information was perceived as highly enabling factor in quality care. The system was found to be quick and easy to use, which engendered staff satisfaction and reportedly improved efficiency. The system was also constructive in providing improved information for resource allocation, which is important in aged care where nurses and care workers were commonly caring for numerous residents at one time.

## 7 RELEVANCE TO CLINICAL PRACTICE

Information systems can aid the organisation and efficiency of nursing work, but current options of digitised nursing care plans in aged care tend to lack convenient integration of both standardised and personalised interventions for residents, and evaluation of satisfaction for residents and staff. This new system offers a positive co‐design that addresses these issues. Importantly, a digital system can be efficient or acceptable if it improves quality first and foremost.

## CONFLICT OF INTEREST

As part of the funding agreement, the funders were able to review manuscript content prior to submission but were unable to withhold consent for publication or demand any changes.

## AUTHOR CONTRIBUTIONS

CRediT author statement Kasia Bail: Conceptualisation, Methodology, Validation, Interviews, Focus Groups, Time and Motion Observations, Formal analysis (quantitative and qualitative), Interpretation, Writing original draft, Writing – Reviewing and Editing, Supervision. Eamon Merrick: Conceptualisation, Methodology, Validation, Formal analysis (quantitative), Writing – Reviewing and Editing. Diane Gibson: Conceptualisation, Methodology, Validation, Time and Motion Observations Formal analysis (qualitative), Writing original draft, Writing – Reviewing and Editing. Karen Strickland: Conceptualisation, Methodology, Validation, Interviews, Focus Groups, Time and Motion Observations, Formal analysis (qualitative), Writing – Reviewing and Editing. Bridget Smith: Conceptualisation, Methodology, Interviews, Focus Groups, Time and Motion Observations, Writing – Reviewing and Editing. Alicia Hind: Conceptualisation, Methodology, Validation, Interviews, Formal analysis (qualitative), Writing – Reviewing and Editing. Catherine Paterson: Time and Motion Observations, Formal analysis (qualitative), Investigation, Writing original draft, Writing – Reviewing and Editing. Maria Kozlovskaia: Conceptualisation, Methodology, Interviews, Focus Groups, Time and Motion Observations, Validation, Writing – Reviewing and Editing. Bernice Redley: Conceptualisation, Methodology, Validation, Formal analysis (quantitative), Investigation, Writing original draft, Writing – Reviewing and Editing, Supervision.

## Supporting information

Supplementary Material

Table S1

## Data Availability

The data that support the findings of this study are available on request from the corresponding author. The data are not publicly available due to privacy or ethical restrictions.
