# Supplementary material for: ‘It enables the carers to see the person first’: Qualitative evaluation of point‐of‐care digital management system in residential aged care
Source: J Clin Nurs. 2022 Mar 14;32(1-2):174–90. doi: 10.1111/jocn.16285 (PMC10078649; doi:10.1111/jocn.16285)
Supplement: Supplementary file 1 — Supplementary Material [file JOCN-32-174-s002.docx]

Supplement 2 – Documentation of Intervention

## Intervention description - Product:

Humanetix product architecture comprises highly configurable micro-services that can be installed as stand-alone products or as a fully integrated suite.  Delivered through the cloud, all data exchanges are TLS encrypted. The underlying data architecture is fact-based, such that any update of data is automatically updated in all dashboards, client summaries, forms and assessments that can be opened in the system.

Humanetix configured the system for aged care (known as Humanetix ACE) in consultation with Jindalee staff resulting in 360 distinct types of clinical activities and 1000 distinct types of resident facts. It replaced a hybrid system comprising a commercial electronic care system supplemented by 60 paper forms staff used to capture information beyond the scope of the electronic system.

Records are made and accessed at each point-of-care using any device. ACE has 18 staff roles, including doctors, nurses, carers, allied health and social workers. All staff use the same real-time, encrypted data and work to each resident’s personalised care plan and schedule.  Data from point-of-care units is collated into dashboards for a “helicopter view” of a facility for management control, including alerts when resident care is overdue.

## Intervention description - Process: The system was implemented in 8 Stages over 12 months, in a staggered design adding additional wings (approximately 8 -30 beds) per stage (See Table 1 and 2). Each Stage was supported with a range of change management and project development strategies (See Table 3).

Each stage (from 1- 8) were facilitated by a combination of internal (Jindalee) and external (Humanetix) staff members.

Roles of core development staff:

- Nurse Educator (Jindalee)
- Deputy Directors of Nursing (Jindalee)
- Operations Manager, Village Services (Jindalee)
- Release Manager (Humanetix)
- Product Owner (Humanetix)
- Lead Test Analysis (Humanetix)
- Business Analysts (Humanetix)

These roles were supported by detailed supportive documentation, including:

1. **End User Documentation**

- the Quick Reference Guide (except for Stage 2)

1. **Release Collateral**

- the Product Release Notes

- the Site Configuration Specification

- the Configuration Release Notes (from Stage 5 onwards)

- List of Care Activities (lists those care activities included/excluded in/from each individual stage)

1. **Training Material (for all stages except 3)**

- the training slide deck

- a Self-Assessment Checklist (for some stages only)

- other training material as applicable, for example:

- Carers scenarios
- Navigation check lists for carers
- Summary list of Clinical paper forms integrated into ACE Care

These documents are commercial-in-confidence and available on request with Humanetix <https://humanetix.com.au/> or lindsay.bevage@humanetix.com.au

Table 1 Key timeline of planned ACE Go Live stages

| **Date To/Commenced** | **Stage** | **Location** | **Bed Numbers** |
| --- | --- | --- | --- |
| March 2019 | Pre-implementation data collection | All wings | 165 |
| 17 July 2019 | Stage 1 Go-live | Lower F Wing only | 8 |
| 16 Oct 2019 | Stage 2 Go-live | Lower F Wing only | 8 |
| 30 Oct 2019 | Stage 2 | Upper F Wing (18 beds) | 26 |
| 6 Nov 2019 | Stage 2 | H Wing (18 beds) | 44 |
| 4 Dec 2019 | Stage 2 | C Wing (33 beds) | 77 |
| 11 Dec 2019 | Stage 3 Go-live | All of the above wings | 77 |
| 23 Jan 2020 | Stage 3 | J Wing (8 beds) | 85 |
| 6 Feb 2020 | Stage 3 | D Wing (7 beds) | 92 |
| 18 Feb 2020 | Stage 4 | A Wing (34 beds) | 126 |
| 18 Feb 2020 | Stage 4 Go-live | All of the above wings | 126 |
| 5 Mar 2020 | Stage 4 | G wing (15 beds) | 141 |
| 19 Aug–4 Sep 2020 | Post-implementation data collection | All wings | 169 |
| 30 August 2020 | Stage 5 | B wing (24 beds) | 169 |
| 24 September 2020 | Stage 6 | All wings | 169 |
| 4 March 2021 | Stage 7 | All wings | 169 |
| 1 July 2021 | Stage 8 | All wings | 169 |

Table 2 Key timeline of events

| **Date** | **UC Evaluation Research Team** | **Humanetix and Jindalee Aged Care** |
| --- | --- | --- |
| Feb 2019 | Presentation of the project to Jindalee and ACE  Staff information packs distributed at Jindalee | SMS about the UC evaluation is sent to all staff members and added in DONs books |
| March 2019 | Pre-implementation data collection commences  Data collection interrupted by the events onsite 12-25 March | Gastroenteritis outbreak in the North wing 12–15 March, Accreditation onsite 12-14 March |
| April 2019 |  | JVS delegation at Jindalee |
| May 2019 | Conducted additional DEMQOL interviews; Conducted data analysis |  |
| Jun 2019 | Conducted additional DEMQOL interviews; Data analysis and Baseline report |  |
| July 2019 | Baseline and Interim report submitted | ACE stage 1 live |
| October 2019 | Documentation collected for QANDAC |  |
| November 2019 |  | ACE stage 2 live |
| December 2019 |  | ACE stage 3 live |
| March 2020 | Post-implementation data collection delayed due to COVID-19 pandemic, and UC protocol  National Cabinet advice to restrict visitation in Aged Care Facilities in response to the COVID-19 pandemic | ACE stage 4 live |
| April - June 2020 | Face-to-face research ceased indefinitely due to COVID-19 pandemic |  |
| July 2020 | UC procedures released for the recommencement of face-to-face research - COVID-19 Project Risk Assessment and Mitigation Plan and Ethics amendment submitted |  |
| August 2020 | Commencement of face-to-face research at Jindalee approved by UC Dean and UC Ethics.  Staff and resident information packs distributed at Jindalee.  Post-implementation data collection commenced in line with COVID-19 precautions | SMS about the UC evaluation is sent to all staff members and added in DONs books  ACE stage 5 live  Roll out of ACE in B-wing |
| September - October 2020 | Documentation analysis |  |
| October - December 2020 | Analysis and report drafting |  |

Table 3. Key timeline of mechanisms for change management and project development

| **Date Commenced** | **Title, duration** | **Time occurrences** | **Key activities** | **Total sessions (as of August 2020)** |
| --- | --- | --- | --- | --- |
| Sep 2018 | Clinical Working Group (CWG) Meetings  (1-2 hours) | Weekly throughout project | User assessment, Planning, Decision making, Feedback. Reviews of Stages  Key staff brought in as required | 70 |
| Feb 2019 | Countdown to Go Live Phases  (1 week) | 1 month before each stage | Trainer training, Testing. Feedback. Updated training material, All staff training, User acceptability test (UAT) Rounds 1 and 2, Preparation of operational environment | 5 |
| Feb 2019 | Training/Feedback sessions (combined)  (1-2 hours) | Weekly/monthly post each Stage implementation | Training Scenarios ‘Scenario of the day’, Form translation, Planned timeframes: Now, soon, later | 22 |
| June 2019 | User Acceptability Testing UAT  (1-2 hours) | 2 meetings prior to each Stage of implementation | Test scenarios with a checklist used to sign off on agreed product for implementation | 15 |
| Feb 2019 | Miscellaneous additional meetings  (1-2 hours) | Ongoing | E.g. Pre-Admission,  Resident data entry, Care schedule,  resident manager workshops | 10 |
| Feb 2019 | Other approaches | Ongoing | Onsite support, “Loggable issues”, post-it note feedback system,  Educator as trainer, trouble-shooter | As needed |
