# Supplementary material for: ‘It enables the carers to see the person first’: Qualitative evaluation of point‐of‐care digital management system in residential aged care
Source: J Clin Nurs. 2022 Mar 14;32(1-2):174–90. doi: 10.1111/jocn.16285 (PMC10078649; doi:10.1111/jocn.16285)
Supplement: Supplementary file 2 — Table S1 [file JOCN-32-174-s001.docx]

**Supplementary Table 1**

Consolidated criteria for reporting qualitative studies (COREQ): 32-item checklist.

| **No** | **Item** | **Guide questions/description** | **Page** |
| --- | --- | --- | --- |
| **Domain 1: Research team and reflexivity** | | |  |
|  | *Personal Characteristics* |  |  |
| 1. | Interviewer/facilitator | Which author/s conducted the interview or focus group? | Title page  P. 5 |
| 2. | Credentials | What were the researcher's credentials? *E.g. PhD, MD* | Title page  p. 5 |
| 3. | Occupation | What was their occupation at the time of the study?  Academic and research staff at University of Canberra | Title page  P. 5. |
| 4. | Gender | Was the researcher male or female?  Field research described above conducted by female researchers. Authorship includes one male. | P5 |
| 5. | Experience and training | What experience or training did the researcher have?  Research qualifications in author details.  All field researchers have postgraduate training and previous experience in field work. | Title Page  Methods P5 |
|  | *Relationship with participants* |  | P5 |
| 6. | Relationship established | Was a relationship established prior to study commencement?  Qualitative data were collected at the end of this two year participatory action research project. The iterative design process involved active relationships between researchers, project staff and management staff of the RACH. All residents and staff received project information packs. | P3  P5 |
| 7. | Participant knowledge of the interviewer | What did the participants know about the researcher? e*.g., personal goals, reasons for doing the research?*  Information packs prepared by the research team were distributed to staff and residents via multiple modes. The packs explained the evaluation project, including project aims, and the research personnel. | P5 |
|  | Interviewer characteristics | What characteristics were reported about the interviewer/facilitator? e.g. *Bias, assumptions, reasons and interests in the research topic*  Data quality, including any potential impact of researcher bias or assumptions, was enhanced by triangulation using data sources (residents, their visitors, care workers, nurses, managers, project staff), and different methods of collection (see above) and by having multiple analysts examining the data independently. The researchers acknowledged their interest as nurse and health researchers to enable evidence focussed aged care. | P5 |
| Domain 2 Study Design | | |  |
|  | *Theoretical framework* |  |  |
| 9. | Methodological orientation and Theory | What methodological orientation was stated to underpin the study? *e.g. grounded theory, discourse analysis, ethnography, phenomenology, content analysis*  Three- stage participatory action research design. | P4 |
|  | *Participant selection* |  |  |
| 10. | Sampling | How were participants selected? *e.g. purposive, convenience, consecutive, snowball*  Purposive and convenience. | P4-5 |
| 11. | Method of approach | How were participants approached? e*.g. face-to-face, telephone, mail, email*  SMS (staff) email (residents and relatives) facility newsletter, posters displayed in the facility. | P4-5 |
| 12. | Sample size | How many participants were in the study?  128 (48 residents or their visitors and 65 staff). | P5 |
| 13. | Non-participation | How many people refused to participate or dropped out? Reasons?  People who declined to participate were not asked for their reasons. Of note is that there was 50% turnover among resident participants (95% due to death) over the study period.  Residents were excluded if unable to communicate effectively or to provide consent at the time (reasons for exclusion included such as sensory deficit, cognitive deficit) | P4 - 5 |
|  | *Setting* |  |  |
| 14. | Setting of data collection | Where was the data collected? e*.g. home, clinic, workplace*  A metropolitan Residential Aged Care Home (RACH) | P3 |
| 15. | Presence of non-participants | Was anyone else present besides the participants and researchers?  Interviews were conducted in bedrooms hallways and common areas where other staff and residents were often present. Focus groups were conducted in a separate room. | p5 |
| 16. | Description of sample | What are the important characteristics of the sample? *e.g. demographic data, date*  The key characteristic of participants for the purposes of this analysis is their role (resident/visitor /RACH staff/Humanetix staff). For RACH staff, a distinction is made between managers, nursing staff, care staff. | P5 |
|  | *Data collection* |  |  |
| 17. | Interview guide | Were questions, prompts, guides provided by the authors? Was it pilot tested?  **Figure 1** provides an example of a topic guide, which was shared with key RACH staff for feedback prior to implementation. | P5, Fig1 |
| 18. | Repeat interviews | Were repeat interviews carried out? If yes, how many?  Data at each cycle were considered by the steering group that included participants. | P3 |
| 19. | Audio/visual recording | Did the research use audio or visual recording to collect the data?  Interview responses captured using iPad or paper field notes. And focus groups were recorded with the permission of the participant. | P5 |
| 20. | Field notes | Were field notes made during and/or after the interview or focus group?  Yes, for the interview and mentioned in the manuscript. | P5 |
| 21. | Duration | What was the duration of the interviews or focus group? | P 4 and 5 |
| 22. | Data saturation | Was data saturation discussed? | P5 |
| 23. | Transcripts returned | Were transcripts returned to participants for comment and/or correction?  Transcripts were not returned, however cycles of development, implementation and feedback as elements of co-design are articulated in the context of the participatory action research design. | P5 |
| **Domain 3: analysis and findings** | | |  |
|  | *Data analysis* |  |  |
| 24. | Number of data coders | How many data coders coded the data?  Analysts included seven researchers and one consumer representative. | P6 |
| 25. | Description of the coding tree | Did authors provide a description of the coding tree?  No, as the study coded on the predetermined study aims as described p6. | P6-12, Table 1 |
| 26. | Derivation of themes | Were themes identified in advance or derived from the data?  Three key evaluation criteria (the Evaluation Framework) were identified prior to project commencement. A fourth criterion relating to the implementation and the role of co-design emerged from the data. | P6,  Table 1 |
| 27. | Software | What software, if applicable, was used to manage the data?  Manual coding was used. | P5 |
| 28. | Participant checking | Did participants provide feedback on the findings?  Participants had the opportunity to provide feedback, suited to their preference of dissemination and discussion in the form of morning and afternoon teas at the RACH, as well as more formally with the Clinical Working Group. | P5 |
|  | *Reporting* |  |  |
| 29. | Quotations presented | Were participant quotations presented to illustrate the themes / findings? Was each quotation identified? e*.g. participant number*  Yes | P6-16  Tables 4-6 |
| 30. | Data and findings consistent | Was there consistency between the data presented and the findings?  Yes | P6-16  Tables 4-6 |
| 31. | Clarity of major themes | Were major themes clearly presented in the findings?  Yes | P6-16  Tables 4-6 |
| 32. | Clarity of minor themes | Is there a description of diverse cases or discussion of minor themes?  Divergent views are included in the data presented | P6-16  Tables 4-6 |

Allison Tong, Peter Sainsbury, Jonathan Craig, Consolidated criteria for reporting qualitative research (COREQ): a 32-item checklist for interviews and focus groups, International Journal for Quality in Health Care, Volume 19, Issue 6, December 2007, Pages 349–357, <https://doi.org/10.1093/intqhc/mzm042>
